# Neuromorphic Analog Implementation of Neural Engineering Framework-Inspired Spiking Neuron for High-Dimensional Representation

**DOI:** 10.3389/fnins.2021.627221

**Published:** 2021-02-22

**Authors:** Avi Hazan, Elishai Ezra Tsur

**Affiliations:** Neuro-Biomorphic Engineering Lab, Department of Mathematics and Computer Science, The Open University of Israel, Ra’anana, Israel

**Keywords:** neural engineering framework, spiking neural networks, neuromorphic electronics, neuromorphic engineering, brain-inspired electronics

## Abstract

Brain-inspired hardware designs realize neural principles in electronics to provide high-performing, energy-efficient frameworks for artificial intelligence. The Neural Engineering Framework (NEF) brings forth a theoretical framework for representing high-dimensional mathematical constructs with spiking neurons to implement functional large-scale neural networks. Here, we present OZ, a programable analog implementation of NEF-inspired spiking neurons. OZ neurons can be dynamically programmed to feature varying high-dimensional response curves with positive and negative encoders for a neuromorphic distributed representation of normalized input data. Our hardware design demonstrates full correspondence with NEF across firing rates, encoding vectors, and intercepts. OZ neurons can be independently configured in real-time to allow efficient spanning of a representation space, thus using fewer neurons and therefore less power for neuromorphic data representation.

## Introduction

Albeit artificial intelligence has emerged as the focal point for countless state-of-the-art developments, in many ways, it is nullified when compared with biological intelligence, particularly in terms of energy efficiency. For instance, the honeybee is capable of exceptional navigation while possessing just under 1 million neurons and consuming only 10^–3^W of power. Comparably, an autonomous car would need to utilize over a 10^3^W of sensing and computing power, demonstrating lamentable energetic efficiency decreased a millionfold (Liu et al., 2014). Consequentially, brain-inspired hardware designs have been used in numerous applications, particularly in neuro-robotics (Krichmar and Wagatsuma, 2011; Zaidel et al., 2021) and smart-edge devices (Krestinskaya et al., 2019; Zhang et al., 2020). In neuromorphic computing architectures, the computational principles of biological neural circuits are utilized to design artificial neural systems. A neuromorphic circuit comprises densely connected, physically implemented computing elements (e.g., silicon neurons), which communicate with spikes (Tsur and Rivlin-Etzion, 2020). Notable neuromorphic hardware includes the TrueNorth (DeBole et al., 2019), developed by IBM research, the Loihi (Davies et al., 2018), developed by Intel Labs, the NeuroGrid (Benjamin et al., 2014), developed at Stanford University, and the SpiNNaker (Furber et al., 2014), developed at the University of Manchester. One theoretical framework, which allows for efficient data encoding and decoding with spiking neurons, is the Neural Engineering Framework (NEF) (Eliasmith and Anderson, 2003). NEF is one of the most utilized theoretical frameworks in neuromorphic computing. It was adopted for various neuromorphic tasks, ranging from neuro-robotics (DeWolf et al., 2020) to high-level cognition (Eliasmith et al., 2012). It was compiled to work on multiple neuromorphic hardware using Nengo, a Python-based “neural compiler,” which translates high-level descriptions to low-level neural models (Bekolay et al., 2014). NEF was shown to be incredibly versatile, as a version of it was compiled on each of the neuromorphic hardware designs listed earlier (Mundy et al., 2015; Boahen, 2017; Fischl et al., 2018; Lin et al., 2018), although they do not follow the same paradigm of neuromorphic implementation. Although the Loihi, the TrueNorth, and the Spinnaker are pure digital systems, in the sense that both computing and communication are held digitally, the NeuroGrid is a mixed analog–digital circuit. In the Neurogrid, synaptic computations were implemented with analog circuitry. Although these general-purpose computing architectures adopted the digital realm for better adherence with application programming and ease of fabrication, analog implementation of synapses (such as the one implemented in the NeuroGrid) is commonly found in analog neuromorphic sensing and signal processing. Notably, some of the first and most significant successes in neuromorphic architectures have been in vision (Indiveri and Douglas, 2000) and sound (Liu and Delbruck, 2010) processing.

NEF-inspired neurons were previously directly implemented in both digital and analog circuitry. For example, NEF-inspired neurons were implemented on a digital Field-Programmable Gate Array (FPGA)-circuit and used for pattern recognition (Wang et al., 2017). However, it is not clear if such implementations can approximate the density, energy efficiency, and resilience of large-scale neuromorphic systems (Indiveri et al., 2011). Current analog implementations of NEF-inspired neurons rely on the circuit fabrication’s stochasticity to constitute the variational activity patterns required to span a representation space. The activity pattern of these neurons cannot be modulated or programmed, and therefore, using them for precise representation of a mathematical construct—even in low dimension—requires a large number of neurons and, hence, has suboptimal energy consumption (see section “Discussion” for further details) (Mayr et al., 2014; Boahen, 2017). Here, we present OZ, a programable, analog implementation of NEF-inspired spiking neuron. OZ utilizes several of the most well-known building blocks for analog spiking neurons to provide a design with a programable high-dimensional response curve and a temporally integrated output.

## Materials and Methods

### Circuit Simulations and Analysis

All circuit simulations in this study were executed using LTspice, offered by Analog Devices (2008). The simulator is based on the open-sourced SPICE framework (Nagel and Pederson, 1973), which utilizes the numerical Newton–Raphson method to analyze non-linear systems (Nichols et al., 1994). Signal analysis was performed using the Python scripts we developed. Curve and surface fittings were performed using MATLAB’s curve fitting toolbox. Simulation files are available upon request.

### Distributed Neuronal Representation With Neural Engineering Framework

Let *a* be a representation, or a function, of a stimulus *x* using *a* = *f*(*x*). With NEF, high-level network specifications, given in terms of vectors and functions, are transformed to a set, or an ensemble, of spiking neurons. A neural representation will therefore take the form of *a* = *G*(*J*(*x*)), where *G* is a spiking neuron model [e.g., the leaky-integrate-and-fire (LIF) model (Burkitt, 2006)] and *J* is the integrated inputs introduced to the neuron. NEF uses a distributed neuron representation, where each neuron *i* responds independently to *x*, resulting in *a*_*i*_ = *G*_*i*_(*J*_*i*_(*x*)). One possible modeling for *J* would be *J* = α*xJ*^*bias*^, where α is a gain term and *J*^*bias*^ is a fixed background current. Neurons often have some preferred stimuli *e* (preferred direction, or encoder) to which they respond with a high frequency of spikes [e.g., direction selectivity in retinal ganglion cells (Ankri et al., 2020)]. *J* will therefore be more appropriately defined using: *J* = α*x*⋅*eJ*^*bias*^, where *x*⋅*e* equals 1 when both *x* and *e* are in the same direction, and 0 when they are opposing each other. To conclude, in NEF, a neuron firing rate δ_*i*_ is defined using:

(1) δi(x)=Gi[αieixJibias]

An ensemble of neurons in which each neuron has a gain and preferred direction distributively represents a vectorized (or high-dimensional) stimulus *x*. The represented stimuli $\hat{x}$ can be decoded using:

(2) $$\hat{x}=\sum_{i}a_{i}*hd_{i}$$

Where *d_i_* is a linear decoder, which was optimized to reproduce *x* using least squared optimization and $a_{i}^{*}h$ is a spiking activity *a_i_*, convolved with a filter *h* (both are functions of time). NEF is described in detail in Eliasmith and Anderson (2003) and succinctly reviewed in Stewart and Eliasmith (2014). NEF is the foundation upon which our neuron model is built. Particularly, it is utilized here to represent a high-dimensional stimulus with spiking neurons distributively.

### Analog Building Blocks

**FIGURE 1:**
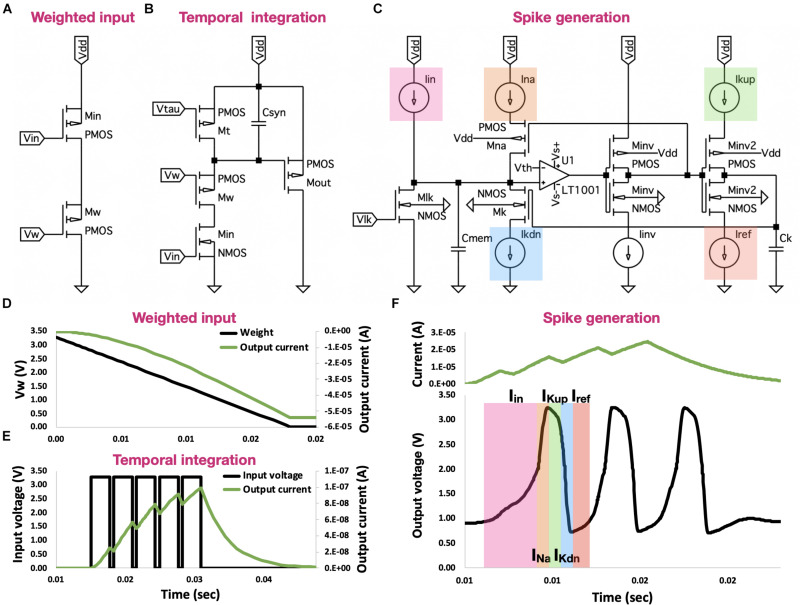
Neuron building blocks. **(A)** Pulse current source synapse. This voltage-controlled current source is activated by an active-low input spike, producing a current, which follows the input voltage pattern and dynamic. **(B)** Subthreshold first-order LPF circuit. This circuit provides temporal control of both charging and discharging of a capacitor, allowing for temporal integration of incoming spikes. **(C)** Voltage-amplifier LIF neuron. This spike generating circuit provides precise control of the generated spikes, including spikes’ rise time, width, fall time, and refractory period. **(D)** Signal traces for the current-source synapse. When spike arrives (synapse is activated-low; *V*_*in*_), current *I*_*syn*_ is proportionally generated. This synapse offers magnitude control where *I*_*syn*_ is proportionally correlated to *V_w_*. **(E)** Signal traces for the log-domain integrator synapse. Log-domain integrator synapse features a linear integration of incoming spikes, where ahead of saturation, each spike equally contributes to *I*_*syn*_. **(F)** Signal traces for the voltage-amplifier LIF neuron. Neuron is driven by *I*_*in*_, which was generated by the subthreshold first-order LPF circuit (described earlier). Circuit has two voltage inverters: first inverter *I*_*inv*_1__ drives current *I*_*NA*_, and second inverter *I*_*inv*_2__ drives the *I_K_* currents. These currents adhere to the behavior of biological neurons, providing precise control of spikes’ dynamic.

In a seminal review by Indiveri et al. (2011) “Neuromorphic silicon neuron circuits,” the fundamental building blocks of analog neurons were described. Among them were (1) the pulse current source synaptic circuit, (2) the subthreshold first-order LPF circuit, and (3) the voltage-amplifier LIF neuron (Figures 1A–C). We will briefly revisit these circuits here, as they constitute the OZ neuron’s main building blocks.

The pulse current-source synapse (Figure 1A), proposed by Mead (1989), was one of the first artificial synapse circuits created. It is a voltage-controlled current source, which is driven by an active-low input spike. The resulting current *I*_*syn*_ is defined using:

(3) $$I_{syn}=I_{o}e^{-\frac{\kappa }{U_{T}}\left(V_{W}-V_{dd}\right)}$$

Where *V*_*dd*_ is the supply voltage, *I_o_* is the leakage current of the transistor *M_w_*, which is activated in the subthreshold regime, κ is the subthreshold slope factor and *U_T_* is a thermal voltage (at room temperature, it is approximately 26 mV). This circuit allows for controlling the magnitude of *I*_*syn*_ such that when *V_w_* equals *V*_*dd*_, *I*_*syn*_ is *I*_0_. As we decrease *V_w_*, *I*_0_ is scaled up exponentially, increasing *I*_*syn*_ accordingly (Figure 1D). While offering control over *I*_*syn*_’s magnitude, the pulse current-source synapse does not provide temporal modulation.

**FIGURE 2:**
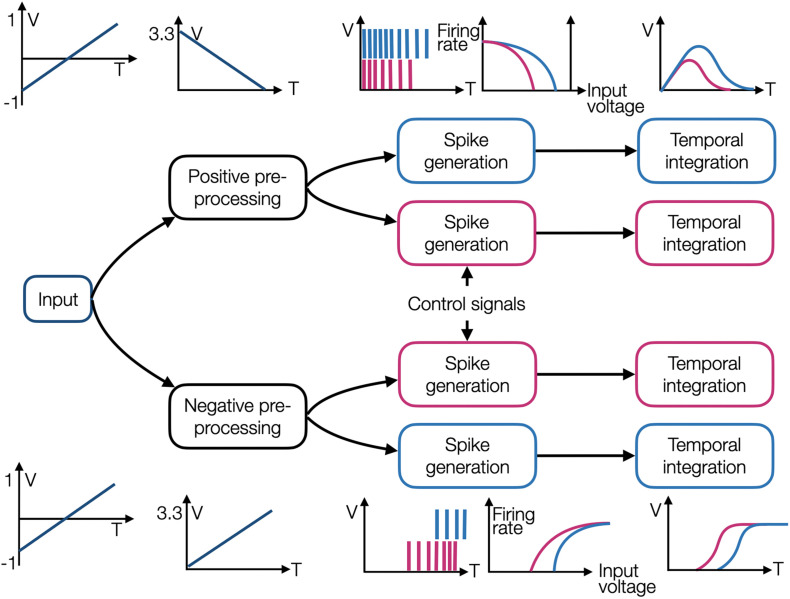
An illustration of four OZ neurons grouped into two branches, one with positive and the other with negative encoders. Each branch initiates with an input preprocessing module, and each neuron comprises a spike generation and temporal integration modules. Each neuron has different tuning, and it is therefore producing spikes in a different dynamic.

The subthreshold first-order LPF circuit (Figure 1B), proposed by Merolla and Boahen (2004), offers linear integration of incoming spikes. This circuit is built upon the charge and discharge synapse [described in Bartolozzi and Indiveri (2007)], which provides temporal control of charging and discharging of *C*_*syn*_. In the charge and discharge synapses, the incoming active-high spikes activate the transistor *M*_*in*_. During a spike, *V*_*syn*_ decreases linearly, at a rate set by the net current *I*_*w*_−*I*_τ_, where *I_w_* is the current driven through transistor *M_w_* (and regulated by *V_w_*) and *I*_τ_ is the current driven through transistor *M*_τ_ (and regulated by *V*_τ_). This net current is responsible for discharging *C*_*syn*_. The linearly decreasing *V*_*syn*_ drives *I*_*syn*_ by regulating transistor *M*_*out*_. In this log-domain circuit, the logarithmic relationship between the transistor’s *V*_*gs*_ and its current is used to exhibit overall linear properties [see (Indiveri et al., 2011) for a detailed analysis]. The governing equations of this synapse during a spike $I_{syn}^{spike}$ and between spikes $I_{syn}^{flat}$ are:

(4) $$I_{syn}^{spike}=\frac{I_{0}}{I_{\tau }}\left(1-e^{-\frac{\left(t-t_{i}^{-}\right)}{\tau_{c}}}\right)I_{syn}^{-}e^{-\frac{\left(t-t_{i}^{-}\right)}{\tau_{c}}}$$

(5) $$I_{syn}^{flat}=I_{syn}e^{-\frac{\left(t-t_{i}^{+}\right)}{\tau_{d}}}$$

where $t_{i}^{-}$ and *t_i_* are the times at which spike *i* arrives and terminates, respectively, $I_{syn}^{-}$ and *I*_*syn*_ are the *I*_*syn*_ in times $t_{i}^{-}$ and *t_i_*, respectively, τ_*c*_ is the time constant for the capacitor charge, which equals *U*_*T*_ = *C*/κ(*I*_*w*_−*I*_τ_), and τ_*d*_ is the time constant for the capacitor-discharge, which equals *U*_*T*_ = *C*/κ*I*_τ_. Controlling the charge and discharge of *C*_*syn*_ allows for temporal control of both rise and fall times of *V*_*syn*_, thus providing the ability to temporally integrate multiple incoming spikes (Figure 2E).

The voltage-amplifier LIF neuron is a spike generating circuit proposed by van Schaik (2001) and Figure 1C. This circuit enhances the classic axon-hillock neuron design [described in Mead (1989)] with precise control of the generated spikes’ dynamic, including spikes’ rise time, width, fall time, and refractory period. Capacitor *C*_*mem*_ models the neuron membrane and *V*_*lk*_, which regulates the conductance of transistor *M*_*lk*_, controls its leakage current *I*_*lk*_. In the absence of an input current from incoming spikes (flat phase), *I*_*lk*_ drives the membrane voltage *V*_*mem*_ down to 0 V. When an input current is apparent, the net incoming current *I*_*in*_−*I*_*lk*_ is charging *C*_*mem*_, increasing *V*_*mem*_. When *V*_*mem*_ exceeds *V*_*th*_, an action potential is generated *via* an operational amplifier (op-amp). This action potential is introduced into a voltage inverter, where high logical states are transformed into low logical states and vice versa. A low logical voltage state activates transistor *M*_*Na*_, through which *I*_*Na*_ current is driven, charging *C*_*mem*_ and creating a sustained high voltage (constituting the spike). A second voltage inverter drives *I*_*kup*_ through transistor *M*_*inv*2_, charging *C_k_*, thus controlling spike’s width. As *C_k_* is charging, it activates transistor *M_k_*, through which *I_k_* is driven. *I_k_* discharges *C*_*mem*_ and when *V*_*mem*_ drops below *V*_*th*_, the amplifier’s output drops to a low state. In response, the first voltage inverter’s output is driven high, deactivating transistor *M*_*Na*_, thus terminating *I*_*Na*_. The second inverter’s output voltage is driven low, terminating *I*_*kup*_ and allowing *I*_*ref*_ to discharge *C_k_*. As long as *I*_*ref*_ is not strong enough to discharge *C_k_*, the circuit cannot be further stimulated by incoming current (assuming *I*_*in*_ < *I*_*k*_), constituting a refractory period. The generated spikes are shown in Figure 1F. This process is a direct embodiment of the biological behavior, in which an influx of sodium ions (Na^+^) and a delayed outflux of potassium ions (K^+^) govern the initiation of an action potential.

## Results

### Circuit Design

**FIGURE 3:**
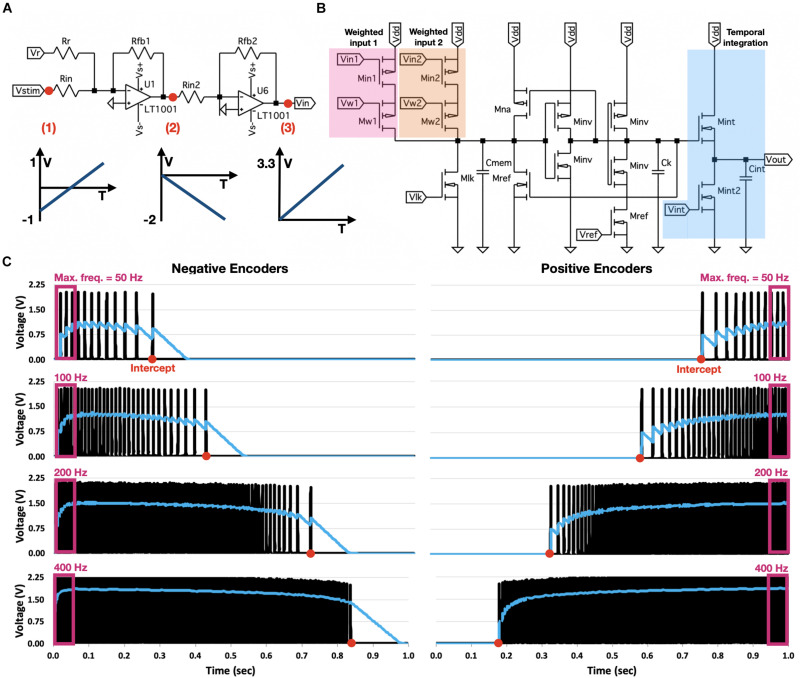
OZ neuron analog design. **(A)** Preprocessing module for negatively encoded OZ neurons. Module inverses input voltage, aligns it to initiate at 0 V, and reinverts and scales it to terminate at 3.3 V. **(B)** Neuron’s spike generator. Voltages from two weighted inputs are transformed into a proportional current, injected into a modified voltage-amplifier LIF neuron. Neuron produces a spike train according to its response dynamic. Spike train is introduced into a temporal integration circuit. **(C)** Eight OZ neurons, four of them are positively encoded (right), and four are negatively encoded (left). All neurons were stimulated with a linearly increasing voltage, rising from –1 to 1 V. Each neuron was modulated with various values of *V*_*lk*_ to produce a spike train at a particular rate, starting from a specific input (intercept).

In our circuit design, stimulus *x* is introduced through preprocessing modules to two branches, one connected to positively encoded OZ neurons and the other to negatively encoded OZ neurons. These preprocessing modules accept an input voltage ranging from −1 to 1 V (corresponding to the default input normalization scheme taken by NEF) and produce an output voltage ranging from 0 to 3.3 V. Each OZ neuron is comprised of two consecutive modules: a spike generator and a temporal integrator. Each spike generator is characterized by a tuning curve, modulated using control signals, thus realizing Eq. 1. A generated spike train is introduced to a temporal integrator, which integrates the incoming spikes, thus realizing Eq. 2 and constituting a NEF-inspired neuron. The circuit schematic for two negatively encoded and two positively encoded neurons is shown in Figure 2. The negative preprocessing circuit comprises two consecutive modules: the first one inverses the voltage and aligns it to initiate at 0 V, and the second reinverts and scales it so it will terminate at 3.3 V (circuit’s*V*_*dd*_) (Figure 3A). The first module uses an op-amp based adder to add 1 V to the input signal (aligning it to 0 V) and inverts it according to *V*_*o*_ = −(*VV*^−^), where *V* and *V*^−^ are the op-amp’s input terminals. The resulted voltage is ranging from 0 to 2 V. The second module uses an inverter amplifier that scales its input voltage according to −*R*_*fb*_/*R*_*in*_, where *R*_*fb*_ is the feedback resistor and *R*_*in*_ is the amplifier’s input terminal resistor. Here, *R*_*fb*2_ = 1.65*kOhm* and *R*_*in*2_ = 1*kOhm*, achieving a scaling factor of −1.65, which transform 2 to 3.3 V output. The positive preprocessing module resembles the negative preprocessing module, with the addition of another voltage inverter, which produces a similar waveform, initiating at 3.3 V and terminating at 0 V.

The OZ neuron is shown in Figure 3B. It is based on modified versions of the pulse current source synaptic circuit (for weighted input), the voltage-amplifier LIF neuron (for spike generation), and the subthreshold first-order LPF circuit (for temporal integration). The pulse-current source synapse is used to convert an input voltage to a proportional current, introduced into the spike generation circuit, and defined by *V_w_* according to Eq. 3. The voltage-amplifier LIF neuron’s response dynamic is predominantly determined by the values of *I*_*kup*_, *I*_*kdn*_, *I*_*Na*_, and *I*_*ref*_ and the leakage current *I*_*lk*_ (driven through transistor *M*_*lk*_), which are regulated, respectively, by of *V*_*kup*_, *V*_*kdn*_, *V*_*Na*_, *V*_*ref*_, and *V*_*lk*_ *via* dedicated transistors. Therefore, this neuron has five degrees of freedom (DOF): *V*_*lk*_ controls the discharge rate of *C*_*mem*_, *V*_*ref*_ controls spikes’ refractory period, *V*_*kup*_ and *V*_*kdn*_ control the fall time of the generated spikes, and *V*_*Na*_ controls the spikes’ rise time. Furthermore, its spiking dynamic relies on an op-amp, which is comprised of multiple transistors and resistors. OZ’s spike generator is a NEF-optimized circuit design, where *V*_*up*_, *V*_*Na*_, and *V*_*kdn*_ are redundant. Furthermore, it does not rely on op-amp for spike generation, as the amplifier has no significant functional effect in terms of neuron’s firing rate and intercept (see section “Discussion”). A NEF-tailored design should also enable high-dimensional input representation, which can be achieved by concatenating the input module (highlighted in Figure 3B as weighted input; see section “Circuit Analysis”). Finally, temporal integration can be achieved *via* a simplified LPF temporal integration circuit. In OZ, capacitor *C*_*int*_ is charged by current *I*_*int*_, which is activated by the generated spike train and driven through transistor *M*_*int*_. *C*_*int*_ is discharging at a constant rate through a leakage current, which is driven through transistor *M*_*int*2_ and regulated by a continuously held voltage *V*_*int*_. The voltage on *C*_*int*_ constitutes the OZ neuron’s output.

A useful way of representing a neuron’s response to varying inputs is by using a response, or a tuning curve, which is one of the most fundamental concepts of NEF. In NEF, a tuning curve is defined using an intercept, the value for which the neuron starts to produce spikes at a high rate, and its maximal firing rate. OZ’s tuning curve can be programmed to control both. For circuit analysis, we built eight OZ neurons, four with positive and four with negative encoders. Each neuron has *d+2* DOF, where *d* is the dimensionality of the input, corresponding to *d* values of *V_w_*, which regulate each input dimension, and *V*_*lk*_ and *V*_*ref*_ correspond to the two other DOF. To demonstrate OZ, we built eight neurons; each was defined to feature a different intercept and maximal firing rate. Each neuron was stimulated with the same input voltage, which linearly increased from −1 to 1 V over 1 s. Results are shown in Figure 3C.

### Circuit Analysis

#### Architectural Design

**FIGURE 4:**
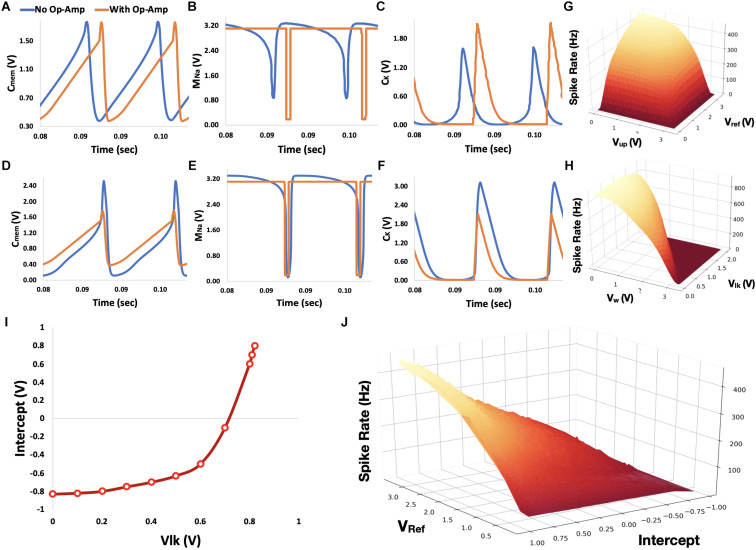
Circuit analysis. **(A–C)** Voltage traces for the subthreshold first-order LPF neuron circuit with and without op-amp for spike generation (colored orange and blue, respectively). Traces for the voltages over *C*_*mem*_, gate of *M*_*Na*_, and *C_K_* are shown in panels **A–C**, respectively. **(D–F)** Similar traced to panels **A–C**, where the transistor-based controls of *I*_*Na*_ and *I*_*kup*_ were eliminated. **(G)** Neuron’s spike rate as a function of *V*_*ref*_ and *V*_*up*_. **(H)** Neuron’s spike rate as a function of *V_w_* and *V*_*lk*_**(I)** Neuron’s intercept as a function of *V*_*lk*_. **(J)** For a given intercept (determined by *V*_*lk*_), a neuron’s spiking rate can be determined by *V*_*ref*_. *V_w_* was held constant at 2.4 V for both **I** and **J** panels.

For the sake of discussion, we will consider one-dimensional (1D) OZ neurons. First, we shall consider the classic voltage-amplifier LIF neuron, shown in Figure 1C. This design relies on an op-amp for spike generation. From a functional perspective, the op-amp provides the neuron with a digital attribute, splitting the neuron into an analog pre-op-amp circuit and a digital post-op-amp circuit. Particularly, when an incoming current is inducing *V*_*mem*_ to exceed a predefined threshold voltage, the op-amp yields a square signal, which generates a sharp *I*_*Na*_ response. This fast response induces sharp swing-ups in *V*_*mem*_ and *V*_*out*_. Without the op-amp, this transition between states is gradual (Figures 4A–C). Although both designs permit spike generation, the op-amp-based design can generate spikes in a higher frequency and amplitude. To compensate for that, we can discard both *I*_*Na*_ and *I*_*kup*_ controls through the removal of their regulating transistors. Removing these resistance-inducing transistors maximizes *I*_*Na*_ and *I*_*kup*_, thus achieving op-amp-like frequency and amplitude (Figures 4D–F). Moreover, without the op-amp, there is no need to explicitly define a threshold, providing a more straightforward and biologically plausible design.

#### Neuron Control

Did we lose control over the maximal firing rate over our neuron by eliminating the regulation of *I*_*kup*_? Fortunately, both *I*_*kup*_ and *I*_*ref*_ impact neuron’s firing rate. Although *I*_*kup*_ limits neuron’s firing rate by governing the rise time of the generated spikes, *I*_*ref*_ does that by setting the refractory period between spikes. Controlling both currents is redundant as both imply similar constraints, as shown in Figure 4G (*V*_*lk*_ and *V_w_* are held constant at 2.2 and 0.5 V, respectively).

*V*_*lk*_ controls the discharge rate of *C*_*mem*_ by regulating a leakage current through transistor *M*_*lk*_. As long as this leakage current is lower than the input current (driven through the weighted input module), *V*_*mem*_ will rise toward saturation. As we decrease *V*_*lk*_, leakage current drops and *C*_*mem*_ charged faster. As a result, the neuron’s intercept (the input value for which the neuron’s initiate spikes at a high rate) increases for positively encoded neurons and decreases for negatively encoded neurons. Neurons exhibit maximal firing rate when their input voltage is either −1 or 1 V, depending on the neuron’s encodings. The maximal firing rate is proportionally dependent on the charging status of membrane capacitance *C*_*mem*_. The faster *C*_*mem*_ is charging, the more frequent the neuron will emit spikes. However, a neuron’s maximal firing rate is not entirely decoupled from its intercept. Although a neuron’s intercept is controlled by *V*_*lk*_, it can also be modulated by *V_w_*, which provides a magnitude control for the input current. Therefore, although *V*_*lk*_ can be used to define the neuron’s intercept, *V_w_* can impose on it a firing rate constraint. For example, the neuron’s spiking rate will not exceed 400 Hz, when *V_w_* is set to 2.2 V. *V*_*lk*_ and *V_w_* imposed constraint on neuron’s spiking rate is demonstrated in Figure 4H (*V*_*ref*_ and *V*_*up*_ are held constant at 3.3 and 1 V, respectively).

Figures 4I,J summarizes the control of the neuron’s intercept and spiking rate using *V*_*ref*_ and *V*_*lk*_ (whereas *V_w_* was held constant at 2.4V). Through curve and surface fittings (*R*^2^ > 0.98), neuron’s intercept *N*_*int*_ can be described with:

(6) $$N_{int}\left(V_{lk}\right)=5.611\cdot 10^{-3}e^{6.911\cdot V_{lk}}-0.8178$$

and neuron’s maximal firing rate *N*_*FR*_ with:

(7) NFR(Vref,Nint)=-36180Vref55Nint-34.4Vref266.3VrefNint

**FIGURE 5:**
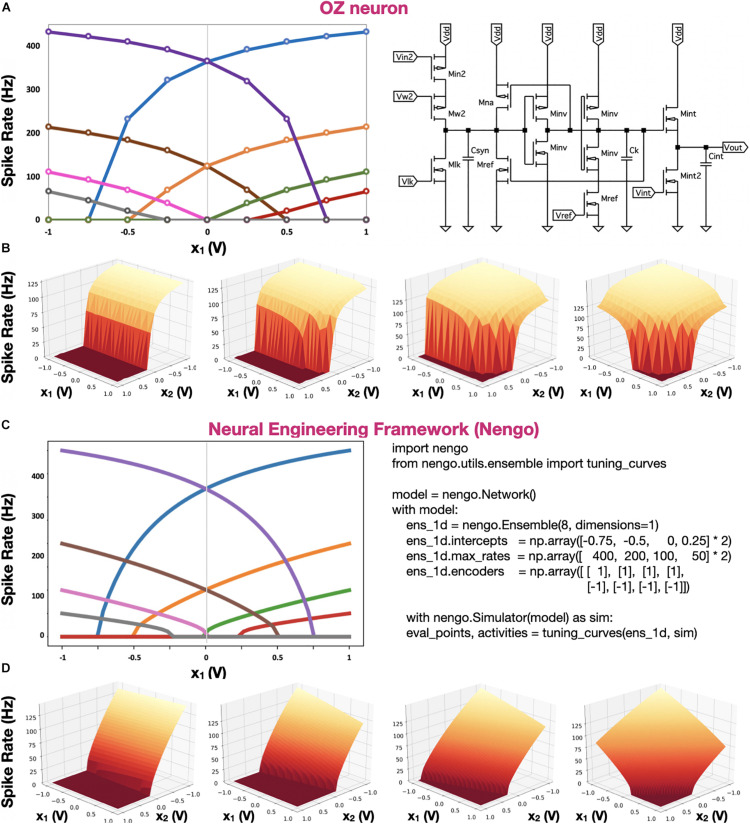
OZ and Nengo neuron tuning curves. **(A)** Tuning curves of eight 1D hardware-based OZ neurons. **(B)** Tuning curves of four 2D hardware-based OZ neurons. **(C)** Tuning curves of eight NEF-based simulated neurons, directly computed with Nengo. **(D)** Four tunning curves of four 2D simulated neurons **(D)**. In panels **A**,**C**, each color stands for one neuron spiking at a specific rate in response to an input voltage (*x*_1_). In OZ-based 2D representation, *V_w_* for *x*_1_ was held constant at 2.5 V, *V*_*ref*_ at 0.4 V, and *V*_*lk*_ at 0.729. *V_w_* values for *x*_2_ were 3.3, 2.8, 2.6, and 2.4 V, left to right, respectively.

In Figure 5, the tuning curves of our eight OZ neurons, along with the tuning curves of eight simulated neurons, which were computed directly with NEF, are demonstrated. The tuning curves indicate varying intercepts and spiking rates, showcasing the produced spike trains’ high predictability and the full correspondence between our hardware design and NEF. In Figures 5B,D, we compared 2D tuning curves. It was achieved with OZ by concatenating two weighted inputs: *x*_1_ and *x*_2_, weighting them with *V*_*w*1_ and *V*_*w*2_, respectively. Results show the high predictability of the neuron in response to a high-dimensional stimulus.

## Discussion

Numerous digital and analog designs of spiking neurons have been previously proposed. For example, Yang et al. (2018b) proposed a biologically plausible, conductance-based implementation of spiking neurons with an FPGA. This design was used to simulate 1 million neurons by utilizing six state-of-the-art FPGA chips simultaneously, achieving biological plausibility and scale (Yang et al., 2018a). Furthermore, it was shown to feature multicompartmental neuron design, supporting the morphologically detailed realization of neural networks (Yang et al., 2019). Biologically plausible spiking neurons were also implemented in analog circuits, featuring spike adaptation (Aamir et al., 2017). Although incredibly versatile and highly configurable, these designs were guided by a bottom–up approach, tailored to reproduce biological behavior. However, to achieve function-optimized neural networks (e.g., for neurorobotics or other smart-edge devices), top–bottom modeling is more suitable (Eliasmith and Trujillo, 2014). By throwing out morphological and physiological constraints, the NEF allows top–down optimization, with which high-level specification can be realized in spiking neurons with a minimal number of explicitly defined neuronal characteristics.

NEF is one of the most utilized theoretical frameworks in neuromorphic computing. A version of NEF was compiled on various neuromorphic digital systems, such as Intel’s Loihi and IBM’s TrueNorth (Fischl et al., 2018; Lin et al., 2018), as well as on hybrid analog/digital systems such as the NeuroGrid (Boahen, 2017). NEF-inspired neurons were directly implemented in both digital (Wang et al., 2017) and analog (Indiveri et al., 2011) circuitry. Although digital NEF-inspired implementations are versatile and programmable, they are fundamentally less energy-efficient and footprint-restricted in comparison with analog circuitry (Amara et al., 2006). Current analog implementations of NEF-inspired neurons rely on the inherent stochasticity in the fabrication process of integrated circuits to create the variational neurons’ tuning required to span a representation space (Mayr et al., 2014; Boahen, 2017) or to support machine learning (Tripathi et al., 2019).

Neurons in NEF represent mathematical constructs, and their accuracy of that representation is fundamentally limited to the neurons’ tuning curves. A tunning curve is defined using an intercept and maximal spike rate. Intercepts represent the part of the representation space for which the neuron will fire. In 1D, uniformly distributed intercepts will uniformly span the representation space. A neuron with an intercept of 0 will be active for 50% of that space, and a neuron with an intercept of 0.75 will be active for only 7.5% of that space. However, using randomly distributed tuning curves would require many more neurons to achieve adequate space spanning. When an input does not invoke a neuron to spike, that neuron is essentially a waste of space and energy.

Moreover, as we advance toward representing values in higher dimensions, articulating and carefully defining neurons’ tuning curves become a critical design factor. This design factor was attested by the authors of Mayr et al. (2014), as they discussed their analog implementation of a NEF-inspired neuron: “as the spread of the curves is determined by random effects of the manufacturing process, individual instances of the ADC [the designated application for that design] have to be checked for sufficient spread, thus defining a yield in terms of ADC resolution. When comparing the two families of tuning curves, the main observation is that the Nengo generated neurons tend to vary more, especially in their gain… this has a significant impact on the overall computation. If the neurons do not encode for sufficiently different features of the input signal, the representation of the input signal degrades” (Mayr et al., 2014).

**FIGURE 6:**
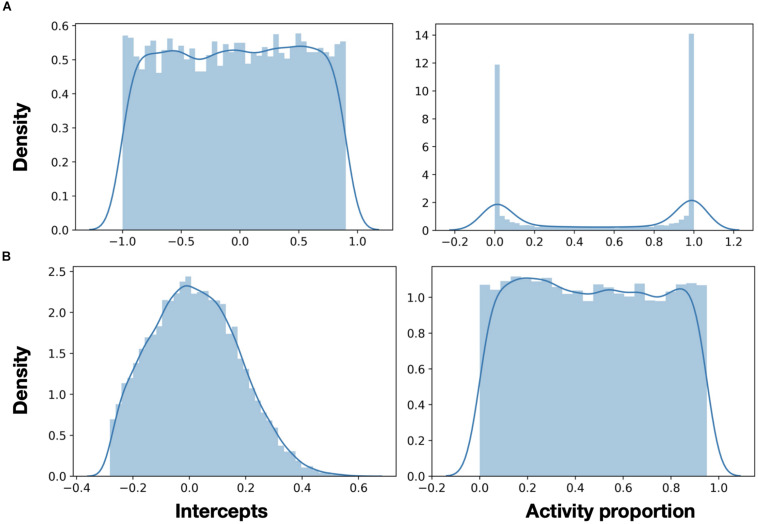
Neurons activity in high dimension. **(A)** In 32D representation, a uniform distribution of intercepts (left) creates many neurons, which are either always or never active (right). **(B)** Using a rational distribution of intercepts (using Eq. 7), a uniform activity pattern of neurons across the representation space can be generated.

Moreover, our proposed implementation offers a high-dimensional representation. Distributing intercepts uniformly between −1 and 1 makes sense for 1D ensembles. Because a neuron’s intercept defines the part of the representation space in which this neuron is firing, in 1D representation, uniformly distributed intercepts create a uniform spanning of that space. In higher dimensions, the proportions of activity are getting smaller (or larger for negatively encoded neurons). In high dimensions, the naive distribution of intercepts results in many neurons, either rarely producing spikes or always active (Figure 6A). In both cases, these neurons are essentially not contributing to the representation. A representation space in 2D is a 3D sphere, in which each neuron’s encoder points to a cap, which specifies the space in which that neuron is active (Gosmann and Eliasmith, 2016). The intercept is the location of the cap’s cutoff plane. The ratio between the cap’s and the sphere’s volumes is the percentage of the representation space in which a neuron is active. A generalized sphere in a higher dimension is a hyper-sphere. The volume of a hyper-sphere cap *v*_*cap*_ is defined with:

(8) $$v_{cap}=\frac{1}{2}C_{d}r^{d}I_{2rh-h^{2}/r^{2}}\left(\frac{d+1}{2},\frac{1}{2}\right)$$

where *C_d_* is the volume of a unit hypersphere of dimension *d* and radius *r*, *h* is the cap’s height, and *I*_*x*_(*a*,*b*) is the regularized incomplete beta function. Here, *r* = 1 (representation is in [−1,1]), and *h* = *x*−1, where *x* is the intercept. The ratio *p* between the hypersphere’s volume *C_d_* and its cap’s volume *v*_*cap*_ is:

(9) $$p=\frac{1}{2}I_{1-x^{2}}\left(\frac{d+1}{2},\frac{1}{2}\right)$$

To more efficiently span in high-dimensional representation space, we can use the inverse of Eq. 6 to derive a desired *p* value, the intercept, which will create it. This equation is defined with:

(10) $$x=\sqrt{1-I_{2p}^{-1}\left(\frac{d+1}{2},\frac{1}{2}\right)}$$

With Eq. 7, we can generate the intersects to better span the representation space. Utilizing this equation can provide the intersects distribution for which the spikes activity pattern is uniform (Figure 6B). This is a clear example of the importance of being able to modulate neuron’s tuning curves in high-dimensional representation. The importance of the discussion earlier was recently highlighted in DeWolf et al. (2020) in the context of neuro-robotics.

Here, we presented the OZ neuron—a programmable analog implementation of a spiking neuron, which can have its tuning curve explicitly defined. With our system design, for a uniform distribution of tuning curves (required in most low-dimensional applications), only one among the positive and negative branches has to be defined, cutting in half the number of neurons, which have to be controlled. Because we can design the neurons’ tuning curve to accurately span the representation space following a particular application’s needs, the required number of neurons for spanning that space can be significantly reduced. Moreover, uniquely, neurons’ tuning curves can be changed in real-time to provide dynamically modulated neuromorphic representation. However, when the required number of neurons is large, the apparent overhead of control must be considered. Our design can be scaled to a full very large-scale integration neuromorphic circuit design, providing analog, distributed, and energy-efficient neuromorphic representation of high-dimensional mathematical constructs.

## Data Availability Statement

Model simulation will be provided upon request.

## Author Contributions

AH designed the circuits and performed circuit simulation and analysis. EE conceptualized the research, designed the circuits, and wrote the manuscript. Both authors contributed to the article and approved the submitted version.

## Conflict of Interest

The authors declare that the research was conducted in the absence of any commercial or financial relationships that could be construed as a potential conflict of interest.

## References

[B1] Aamir S. A. Muller P. Kriener L. Kiene G. Schemmel J. Meier K. (2017). “From LIF to AdEx neuron models: accelerated analog 65 nm CMOS implementation,” in *Proceedings of the IEEE Biomedical Circuits and Systems Conference (BioCAS)*, Turin. 10.1109/BIOCAS.2017.8325167

[B2] Amara A. Amiel F. Ea T. (2006). FPGA vs. ASIC for low power applications. *Microelectron. J.* 37 669–677. 10.1016/j.mejo.2005.11.003

[B3] Analog Devices (2008). *LTspice simulator.* Available online at: http://www.analog.com/en/design-center/design-tools-and-calculators/ltspice-simulator.html (accessed September 26, 2020).

[B4] Ankri L. Ezra-Tsur E. Maimon S. R. Kaushansky N. Rivlin-Etzion M. (2020). Antagonistic center-surround mechanisms for direction selectivity in the retina. *Cell Rep.* 31:107608. 10.1016/j.celrep.2020.107608 32375036 PMC7221349

[B5] Bartolozzi C. Indiveri G. (2007). Synaptic dynamics in analog VLSI. *Neural Comput.* 19 2581–2603. 10.1162/neco.2007.19.10.2581 17716003

[B6] Bekolay T. Bergstra J. Hunsberger E. DeWolf T. Stewart T. Rasmussen D. (2014). Nengo: a Python tool for building large-scale functional brain models. *Front. Neuroinform.* 7:48. 10.3389/fninf.2013.00048 24431999 PMC3880998

[B7] Benjamin B. V. Gao P. McQuinn E. Choudhary S. Chandrasekaran A. Bussat J.-M. (2014). Neurogrid: a mixed-analog-digital multichip system for large-scale neural simulations. *Proc. IEEE* 102 699–716. 10.1109/JPROC.2014.2313565

[B8] Boahen K. (2017). A neuromorph’s prospectus. *Comput. Sci. Eng.* 19 14–28. 10.1109/MCSE.2017.33

[B9] Burkitt A. (2006). A review of the integrate-and-fire neuron model: I. Homogeneous synaptic input. *Biol. Cybern.* 95 1–19. 10.1007/s00422-006-0068-6 16622699

[B10] Davies M. Srinivasa N. Lin T.-H. Chinya G. Cao Y. Choday S. H. (2018). Loihi: a neuromorphic manycore processor with on-chip learning. *IEEE Micro* 38 82–99. 10.1109/MM.2018.112130359

[B11] DeBole M. V. Taba B. Amir A. Akopyan F. Andreopoulos A. Risk W. P. (2019). TrueNorth: accelerating from zero to 64 million neurons in 10 years. *Computer* 52 20–29. 10.1109/MC.2019.2903009

[B12] DeWolf T. Jaworski P. Eliasmith C. (2020). Nengo and low-power AI hardware for robust, embedded neurorobotics. *arXiv* [Preprint]. arXiv: 2007.10227 10.3389/fnbot.2020.568359 33162886 PMC7581863

[B13] Eliasmith C. Anderson C. H. (2003). *Neural engineering: Computation, Representation, and Dynamics in Neurobiological Systems.* Cambridge, MA: MIT press.

[B14] Eliasmith C. Stewart T. C. Choo X. Bekolay T. DeWolf T. Tang Y. (2012). A large-scale model of the functioning brain. *Science* 338 1202–1205. 10.1126/science.1225266 23197532

[B15] Eliasmith C. Trujillo O. (2014). The use and abuse of large-scale brain models. *Curr. Opin. Neurobiol.* 25 1–6. 10.1016/j.conb.2013.09.009 24709593

[B16] Fischl K. Andreou A. Stewart T. Fair K. (2018). “Implementation of the neural engineering framework on the TrueNorth neurosynaptic system,” in *Proceedings of the IEEE Biomedical Circuits and Systems Conference (BioCAS)*, Cleveland, OH. 10.1109/BIOCAS.2018.8584720

[B17] Furber S. Galluppi F. Temple S. Plana L. A. (2014). The spinnaker project. *Proc. IEEE* 102 652–665. 10.1109/JPROC.2014.2304638

[B18] Gosmann J. Eliasmith C. (2016). Optimizing semantic pointer representations for symbol-likeprocessing in spiking neural networks. *PloS One* 11:e0149928. 10.1371/journal.pone.0149928.g006 PMC4762696 26900931

[B19] Indiveri G. Douglas R. (2000). Neuromorphic vision sensors. *Science* 288 1189–1190. 10.1126/science.288.5469.1189 10841740

[B20] Indiveri G. Linares-Barranco B. Hamilton T. J. van Schaik A. Etienne-Cummings R. Delbruck T. (2011). Neuromorphic silicon neuron circuits. *Front. Neurosci.* 5:73. 10.3389/fnins.2011.00073 21747754 PMC3130465

[B21] Krestinskaya O. James A. P. Chua L. (2019). Neuromemristive circuits for edge computing: a review. *IEEE Trans. Neural Netw. Learn. Syst.* 31 4–23. 10.1109/TNNLS.2019.2899262 30892238

[B22] Krichmar J. L. Wagatsuma H. (2011). *Neuromorphic and Brain-Based Robots.* Cambridge: Cambridge University Press. 10.1017/CBO9780511994838

[B23] Lin C.-K. Wild A. Chinya G. Cao Y. Davies M. Lavery D. M. (2018). Programming spiking neural networks on intel’s loihi. *Computer* 51 52–61. 10.1109/MC.2018.157113521

[B24] Liu S.-C. Delbruck T. (2010). Neuromorphic sensory systems. *Curr. Opin. Neurobiol.* 20 288–295. 10.1016/j.conb.2010.03.007 20493680

[B25] Liu S.-C. Delbruck T. Indiveri G. Whatley A. Douglas R. (2014). *Event-Based Neuromorphic Systems.* Hoboken, NJ: John Wiley & Sons. 10.1002/9781118927601

[B26] Mayr C. Partzsch J. Noack M. Schuffny R. (2014). Configurable analog-digital conversion using the neural engineering framework. *Front. Neurosci.* 8:201. 10.3389/fnins.2014.00201 25100933 PMC4106401

[B27] Mead C. (1989). *Analog VLSI and Neural Systems.* Reading, MA: Addison-Wesley Longman Publishing.

[B28] Merolla P. Boahen K. (2004). “A recurrent model of orientation maps with simple and complex cells,” in *Proceedings of the Advances in Neural Information Processing Systems 16*, eds Thrun S. Saul L. (Cambridge, MA: MIT Press), 995–1002.

[B29] Mundy A. Knight J. S. T. Furber S. (2015). “An efficient SpiNNaker implementation of the neural engineering framework,” in *Proceedings of the International Joint Conference on Neural Networks (IJCNN)*, Killarney. 10.1109/IJCNN.2015.7280390

[B30] Nagel L. Pederson D. (1973). *SPICE (Simulation Program With Integrated Circuit Emphasis)* Technical Report No. UCB/ERL M382 April 1973. Berkeley, CA: University of California.

[B31] Nichols K. Kazmierski T. Zwolinski M. Brown A. (1994). Overview of SPICE-like circuit simulation algorithms. *IEE Proc. Circuits Devices Syst.* 141 242–250. 10.1049/ip-cds:19941246 19941246

[B32] Stewart T. Eliasmith C. (2014). Large-scale synthesis of functional spiking neural circuits. *Proc. IEEE* 102 881–898. 10.1109/JPROC.2014.2306061

[B33] Tripathi A. Arabizadeh M. Khandelwal S. Thakur C. S. (2019). “Analog Neuromorphic System Based on Multi Input Floating Gate MOS Neuron Model,” in *Proceedings of the IEEE International Symposium on Circuits and Systems (ISCAS)*, Sapporo. 10.1109/ISCAS.2019.8702492

[B34] Tsur E. E. Rivlin-Etzion M. (2020). Neuromorphic implementation of motion detection using oscillation interference. *Neurocomputing* 374 54–63. 10.1016/j.neucom.2019.09.072

[B35] van Schaik A. (2001). Building blocks for electronic spiking neural networks. *Neural Netw.* 6 617–628. 10.1016/S0893-6080(01)00067-3 11665758

[B36] Wang R. Thakur C. S. Cohen G. Hamilton T. J. Tapson J. van Schaik A. (2017). Neuromorphic hardware architecture using the neural engineering framework for pattern recognition. *IEEE Trans. Biomed. Circuits Syst.* 11 574–584. 10.1109/TBCAS.2017.2666883 28436888

[B37] Yang S. Deng B. Wang J. Li H. Lu M. Che Y. (2019). Scalable digital neuromorphic architecture for large-scale biophysically meaningful neural network with multi-compartment neurons. *IEEE Trans. Neural Netw. Learn. Syst.* 31 148–162. 10.1109/TNNLS.2019.2899936 30892250

[B38] Yang S. Wang J. Deng B. Liu C. Li H. Fietkiewicz C. (2018a). Real-time neuromorphic system for large-scale conductance-based spiking neural networks. *IEEE Trans. Cybern.* 49 2490–2503. 10.1109/TCYB.2018.2823730 29993922

[B39] Yang S. Wang J. Lin Q. Deng B. Wei X. Liu C. (2018b). Cost-efficient FPGA implementation of a biologically plausible dopamine neural network and its application. *Neurocomputing* 314 394–408. 10.1016/j.neucom.2018.07.006

[B40] Zaidel Y. Shalumov A. Volinski A. Supic L. Ezra Tsur E. (2021). Neuromorphic NEF-based inverse kinematics and PID control. *Front. Neurorobot.* 15:631159. 10.3389/fnbot.2021.631159 PMC7887770 33613225

[B41] Zhang W. Gao B. Tang J. Yao P. Yu S. Chang M.-F. (2020). Neuro-inspired computing chips. *Nat. Electron.* 3 371–382. 10.1038/s41928-020-0435-7

